# Inhibition of lignin-derived phenolic compounds to cellulase

**DOI:** 10.1186/s13068-016-0485-2

**Published:** 2016-03-22

**Authors:** Lei Qin, Wen-Chao Li, Li Liu, Jia-Qing Zhu, Xia Li, Bing-Zhi Li, Ying-Jin Yuan

**Affiliations:** Key Laboratory of Systems Bioengineering (Ministry of Education), School of Chemical Engineering and Technology, Tianjin University, Weijin Road 92, Nankai District, Tianjin, 300072 People’s Republic of China; SynBio Research Platform, Collaborative Innovation Center of Chemical Science and Engineering (Tianjin), Tianjin University, Weijin Road 92, Nankai District, Tianjin, 300072 People’s Republic of China

**Keywords:** Phenolics, Vanillin, Inhibition, Cellulose, Enzymatic hydrolysis, Cellulase

## Abstract

**Background:**

Lignin-derived phenolic compounds are universal in the hydrolysate of pretreated lignocellulosic biomass. The phenolics reduce the efficiency of enzymatic hydrolysis and increase the cost of ethanol production. We investigated inhibition of phenolics on cellulase during enzymatic hydrolysis using vanillin as one of the typical lignin-derived phenolics and Avicel as cellulose substrate.

**Results:**

As vanillin concentration increased from 0 to 10 mg/mL, cellulose conversion after 72-h enzymatic hydrolysis decreased from 53 to 26 %. Enzyme deactivation and precipitation were detected with the vanillin addition. The enzyme concentration and activity consecutively decreased during hydrolysis, but the inhibition degree, expressed as the ratio of the cellulose conversion without vanillin to the conversion with vanillin (*A*_*0*_/*A*), was almost independent on hydrolysis time. Inhibition can be mitigated by increasing cellulose loading or cellulase concentration. The inhibition degree showed linear relationship with the vanillin concentration and exponential relationship with the cellulose loading and the cellulase concentration. The addition of calcium chloride, BSA, and Tween 80 did not release the inhibition of vanillin significantly. pH and temperature for hydrolysis also showed no significant impact on inhibition degree. The presence of hydroxyl group, carbonyl group, and methoxy group in phenolics affected the inhibition degree.

**Conclusion:**

Besides phenolics concentration, other factors such as cellulose loading, enzyme concentration, and phenolic structure also affect the inhibition of cellulose conversion. Lignin-blocking agents have little effect on the inhibition effect of soluble phenolics, indicating that the inhibition mechanism of phenolics to enzyme is likely different from insoluble lignin. The inhibition of soluble phenolics can hardly be entirely removed by increasing enzyme concentration or adding blocking proteins due to the dispersity and multiple binding sites of phenolics than insoluble lignin.

**Electronic supplementary material:**

The online version of this article (doi:10.1186/s13068-016-0485-2) contains supplementary material, which is available to authorized users.

## Background

Biorefinery of lignocellulosic biomass to liquid fuels or other chemicals is beneficial to sustainable energy and environment [1]. Lignocellulose is mainly composed of cellulose, hemicellulose, and lignin. Cellulose and hemicellulose can be converted to fermentable sugars by enzymatic hydrolysis, while lignin plays a negative role on the saccharification of lignocellulosic biomass [2]. Lignin is an aromatic polymer consisting of three primary units: hydroxyphenyl (H), guaiacyl (G), and syringyl (S) units, which are randomly linked with aryl ether, ester, or carbon bonds. Lignin adheres the carbohydrates together and strengthens the cell wall structure to give rise to biomass recalcitrance [3–5].

Pretreatment is requisite to break the biomass recalcitrance by changing chemical or physical properties of biomass and thus increase the enzyme accessibility to cellulose [6, 7]. Delignification not only enhances cellulose digestibility by increasing the accessibility of cellulose but also reduces the adsorption of cellulase on lignin [8–10]. Lignin inhibition caused by non-productive enzyme adsorption has been deeply studied in the past few years [11–14]. During the pretreatment, lignin is generally degraded into kinds of phenolics or oligomers. Both complete lignin [8–14] and soluble lignin derivatives (phenolics) [15–17] after pretreatment process hamper enzyme hydrolysis and reduce sugar yields. Understanding and reducing the inhibition of phenolics in enzymatic hydrolysis are an important issue to improve the efficiency of bioconversion of lignocellulose.

Soluble phenolics are generally generated during most pretreatment processes, such as acidic or alkaline pretreatment, regardless of herbage, softwood or hardwood [18, 19]. The type and content of soluble phenolics are up to the biomass species and the pretreatment methods, and phenolics concentration also depends on the solid loading in pretreatment and enzymatic hydrolysis. Dry-to-dry pretreatment process (e.g., AFEX and ethylenediamine pretreatment) [20, 21] saves water usage as well as increases soluble phenolics concentration in the subsequent enzymatic hydrolysis. Increasing solid loading in enzymatic hydrolysis also increases the phenolics concentration significantly [22]. Previous studies supposed that the degraded lignin is less harmful to enzymes than macromolecular lignin [23, 24]. However, phenolics are much more inhibitory than soluble sugars, furan derivatives, and organic acids, as phenolics can lead to precipitation and irreversible inhibition of enzymes [13, 17]. Furthermore, cellulase is found more susceptible to be inhibited than *β*-glucosidase [13, 15]. Inhibition degree of phenolics depends on phenolics concentration with linear correlation or non-linear correlations [25, 26]. However, our knowledge about inhibition of phenolics is still limited to overcome the problem.

In this study, a commercial enzyme Spezyme CP containing exo-, endo-cellulase, and *β*-glucosidase activities was used to examine the effect of phenolics on cellulase mixture. We used pure cellulose (Avicel) as substrate to avoid the interference of other compositions in lignocellulosic materials. Vanillin was employed as model phenolic, due to its common existence in pretreated lignocellulose [18, 22]. We investigated the effects of cellulose concentration, cellulase concentration, pH, and temperature on the inhibition of phenolics to enzymatic activity. Furthermore, we tried to reduce phenolics inhibition by adding non-active proteins and surfactants. The fundamental insights would be constructive to understand the mechanism of phenolics inhibition and improve the enzymatic hydrolysis of lignocellulosic biomass.

## Results and discussion

### Phenolics denature and inhibit cellulose during enzymatic hydrolysis of cellulose

**Fig. 1:**
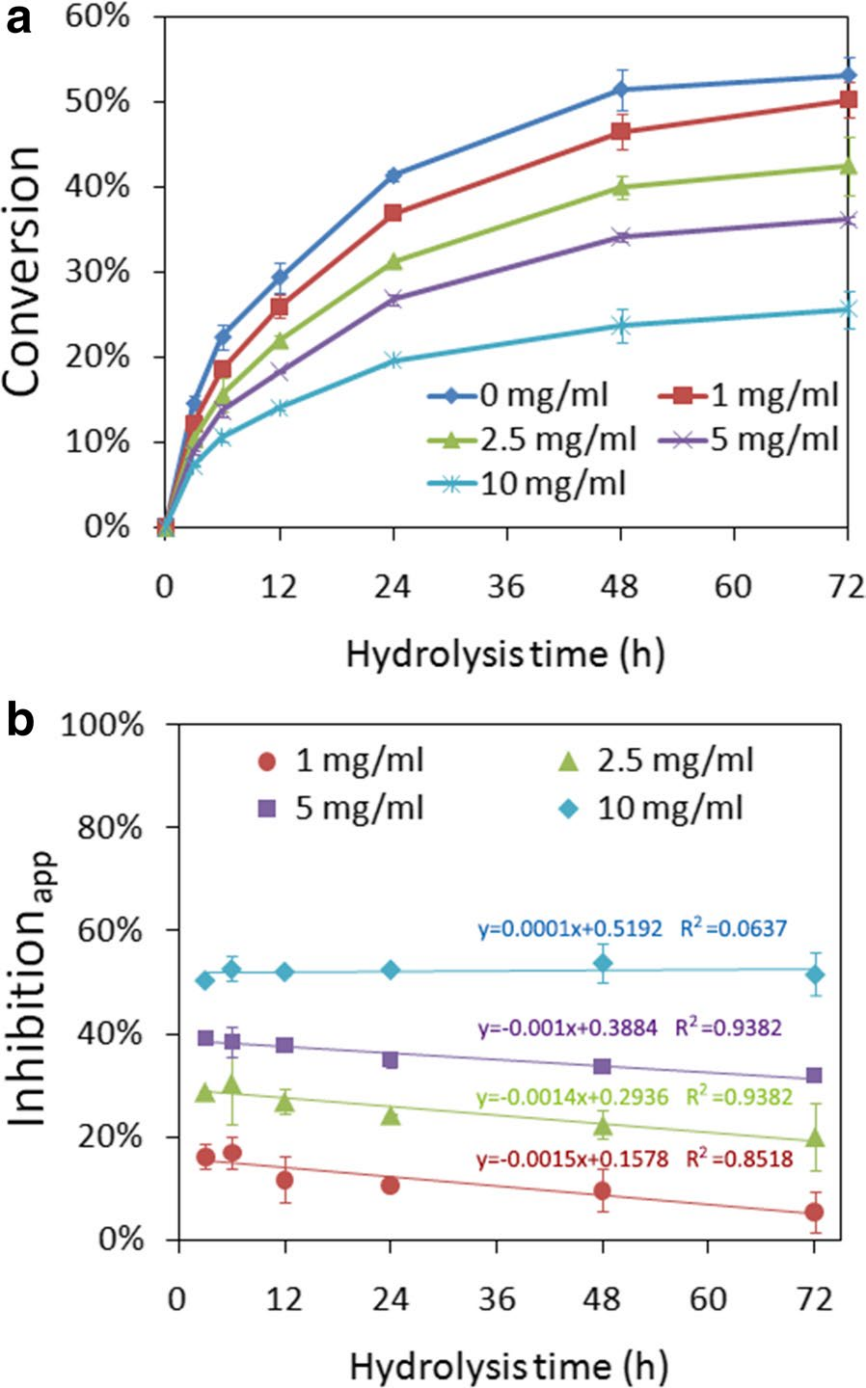
Effect of vanillin concentration on enzymatic hydrolysis (**a**) and apparent inhibition degrees (**b**) during cellulose conversion. Cellulose loading was 1 % (10 mg/mL) and cellulase concentration was 0.3 mg/mL. *Error bars* represented standard deviations, *n* = 3

As vanillin is a main phenolic compound from lignin degradation, we studied the effect of different vanillin concentrations on cellulose conversion catalyzed by a commercial cellulase (Spezyme CP) in hydrolysate (Fig. 1). Our study indicated that the phenolics concentration in the hydrolysate of ethylenediamine pretreated corn stover with high solid loading reaches 10 mg/mL. The addition of 1–10 mg/mL vanillin resulted in decreased cellulose conversions (Fig. 1a) and decreased hydrolysis rate (Additional file 1: Figure S1). We further calculated apparent inhibition degree (Inhibition_app_) based on the control (cellulose conversion without inhibitor addition) as following equation:

1 $$ {\text{Inhibition}}_{\text{app}} = \, 1 \, - \, A/A_{0} $$

where *A* is cellulose conversion with inhibitor at specific hydrolysis time; *A*_*0*_ is cellulose conversion without inhibitor at the same hydrolysis time and the same condition with *A*.

It is obvious that inhibition_app_ is nearly a constant at 10 mg/mL vanillin (within a range from 50 to 54 %), and inhibition_app_ slightly decreased at lower vanillin concentrations (1–5 mg/mL) along with the hydrolysis time (e.g., inhibition_app_ of 5 mg/mL vanillin decreased from 39 to 32 % within 72 h) (Fig. 1b). The decrease of inhibition_app_ at lower inhibitor concentration may be ascribed to the product inhibition by glucose and cellubiose, because the glucose and cellubiose accumulated to a high concentration at the later hydrolysis stages [22].

Enzyme concentrations in supernatant at different conditions were determined (Additional file 2: Figure S2). Without cellulose substrate addition, cellulase concentration decreased by 42 % after 72-h incubation in citrate buffer under the same condition of enzymatic hydrolysis (50 °C, pH 4.8), which is due to the thermal denaturation of enzymes. The enzyme activity at different incubation times also decreased, consistent with enzyme concentration (Additional file 3: Figure S3). In the real enzymatic hydrolysis, enzyme concentration was lower than that without substrate in initial 3 h due to the productive adsorption in substrate. However, the enzyme concentration with substrate became higher than that without substrate after 24 h (Additional file 2: Figure S2). This result revealed that the binding of enzymes and substrate benefits to maintain the enzyme activity. In addition, with the addition of vanillin, cellulase concentration after 72 h decreased by 58 and 54 % for that with and without substrate addition, respectively, which indicated that vanillin accelerated the denaturation of cellulase (Additional file 2: Figure S2). The enzyme activity with vanillin addition decreased from 45 to 13 % from 24 to 72 h (Additional file 3: Figure S3), while the enzyme concentration only decreased from 0.17 to 0.14 mg/mL at the same time (Additional file 2: Figure S2). This inconsistence of enzyme activity and enzyme concentration implied the accessional inhibition of vanillin on cellulase activity apart from protein denaturation. Inhibition of phenolics on cellulase in hydrolysate was a complicated course, including irreversible inhibition and reversible inhibition simultaneously [13].

### Effect of cellulase concentration and cellulose loading on inhibition

**Fig. 2:**
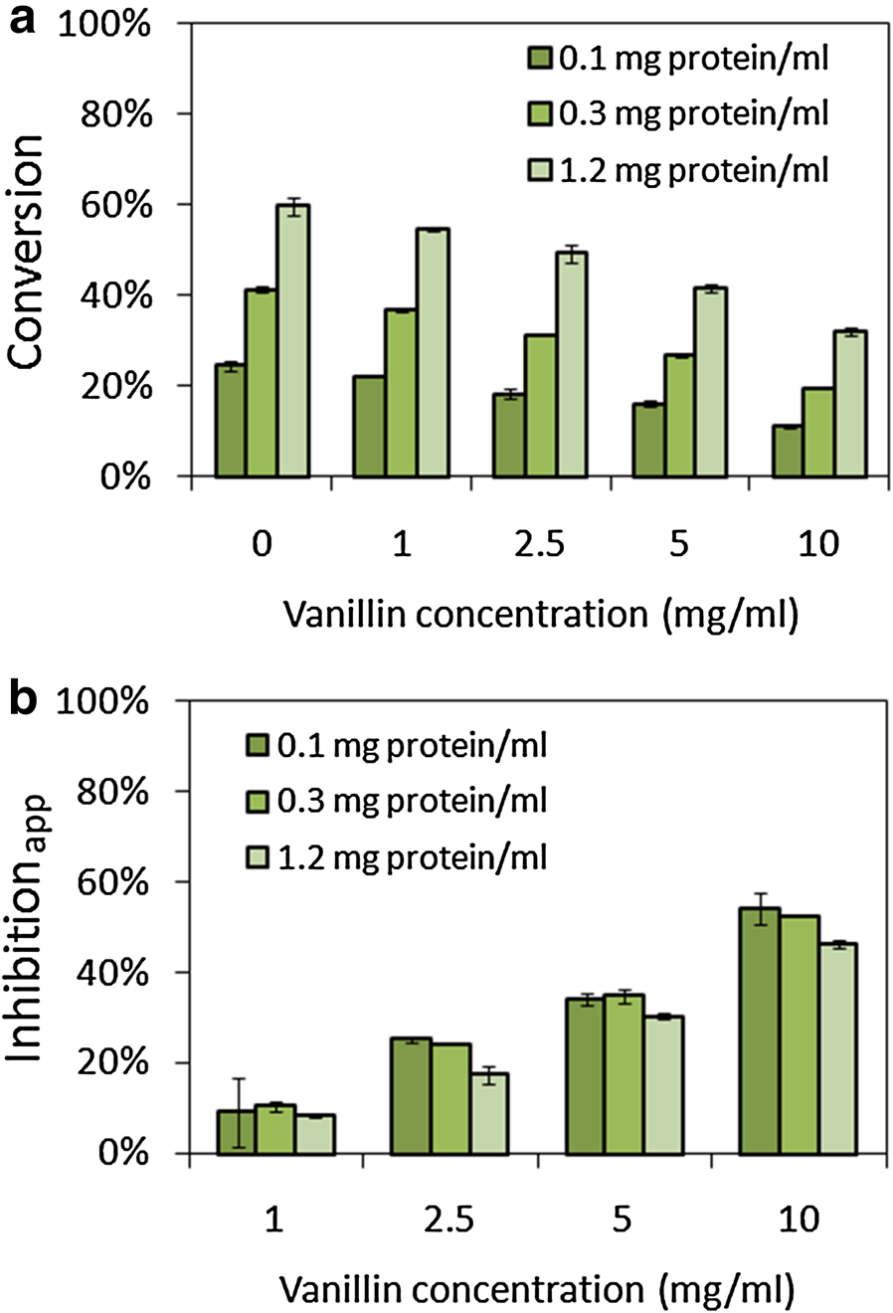
Effect of enzyme concentration on cellulose conversions (**a**) and apparent inhibition (**b**) at 24 h of hydrolysis. Cellulose loading was 1 %. *Error bars* represented standard deviations, *n* = 3

**Fig. 3:**
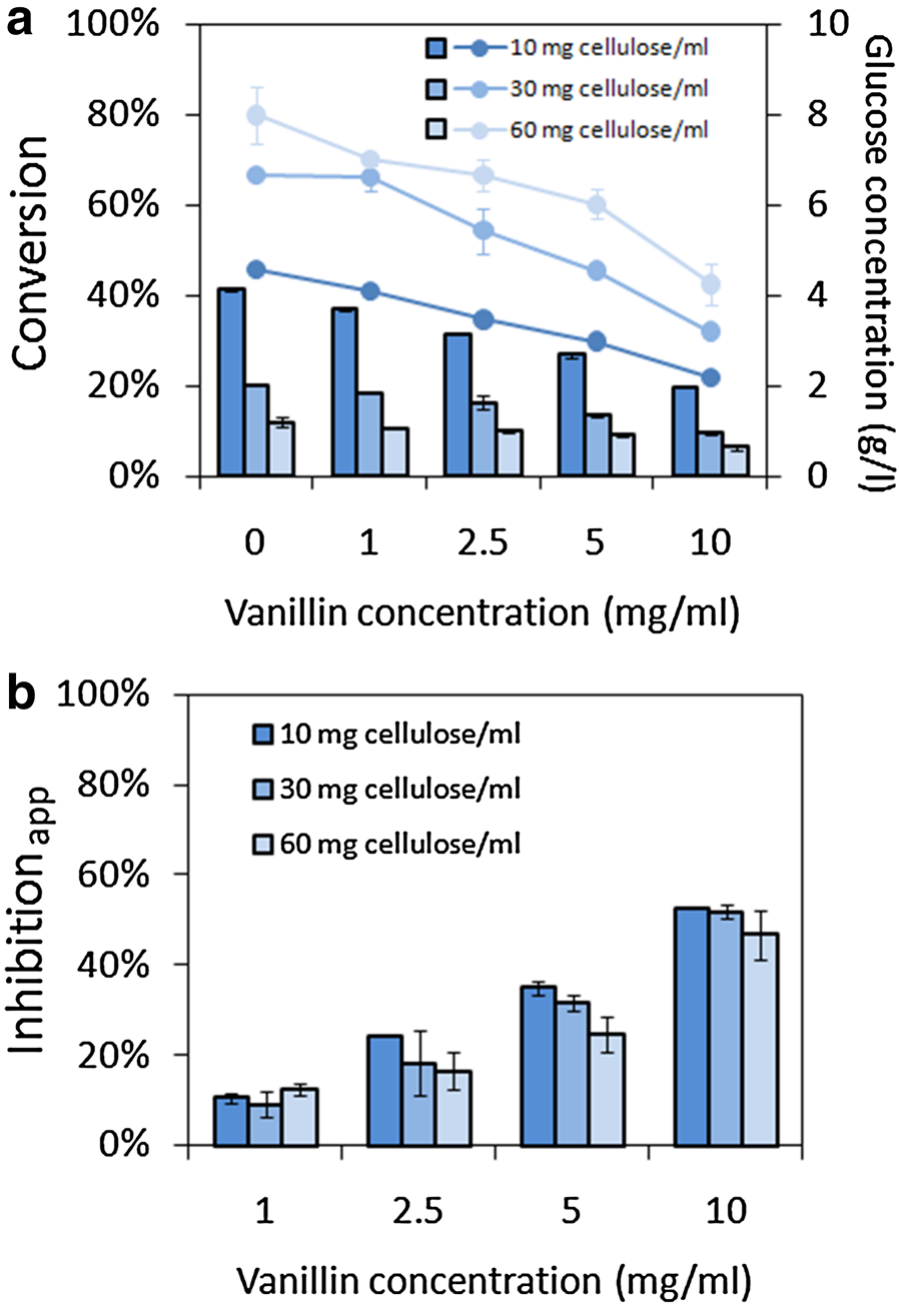
Effect of cellulose concentration on cellulose conversions (**a**) and apparent inhibition (**b**) at 24 h of hydrolysis. *Scatter plots* represent glucose concentration and histograms represent cellulose conversion in (**a**). Cellulase concentration was 0.3 mg/ml. *Error bars* represented standard deviations, *n* = 3

As previously speculated, increasing cellulase loading may relieve the inhibition of phenolics [27]. We investigated the inhibition of vanillin on cellulose hydrolysis with different enzyme concentrations (Fig. 2). Cellulose conversion obviously increased as enzyme concentration increasing (Fig. 2a). However, as the function (1) defined, the apparent inhibitions decreased slightly along with the increasing enzyme concentration (Fig. 2b). For example, at 10 mg/mL vanillin, inhibition_app_ only decreased from 54 to 46 % when enzyme concentration increased from 0.1 to 1.2 mg/mL. These results indicated that increasing cellulase concentration slightly reduced inhibition_app_. The higher enzyme concentration than this range was not studied here. The effect of cellulose concentration on remission of vanillin inhibition was also investigated (Fig. 3). The subtle decrease of inhibition_app_ was also observed by increasing cellulose concentration except for 1 mg/mL vanillin (Fig. 3b). The reduction of inhibition_app_ was not significant at 1 mg/mL vanillin.

As enzyme concentration, cellulose concentration and inhibitor concentration proportionally increased, inhibition significantly increased (Additional file 4: Figure S4), which indicated that comparing to the increasing of enzyme concentration or cellulose concentration, the increasing of inhibitor concentration was more significant to affect the inhibition degree. This result proves that the declined cellulose conversion with higher solid loading in enzymatic hydrolysis is attributed to the increasing inhibitor concentration in many reports [22, 28].

Although the remission of inhibition by increasing enzyme concentration and cellulose concentration is limited, the reason for this tiny remission was unknown. It was proposed that the equilibrium between adsorption of enzyme on cellulose and the denaturation of enzyme by inhibitor would regulate the effect of enzyme concentration and cellulose concentration on the inhibition. Inhibitor-binding constant [25] was employed to analyze the results. In the studies of reversible inhibition from glucose and cellubiose, the ratio of cellulose conversion without inhibitor to that with inhibitor (*A*_*0*_/*A*) has a linear relationship with inhibitor concentration as following expression:

2 $$ A_{0} /A \, = \, 1 \, + \beta \, I $$

where *I* is the concentration of inhibitor (g/L); *β* is the inhibitor-binding constant (L/g). The higher *β* value means the stronger combining capacity of inhibitor to enzyme.

**Fig. 4:**
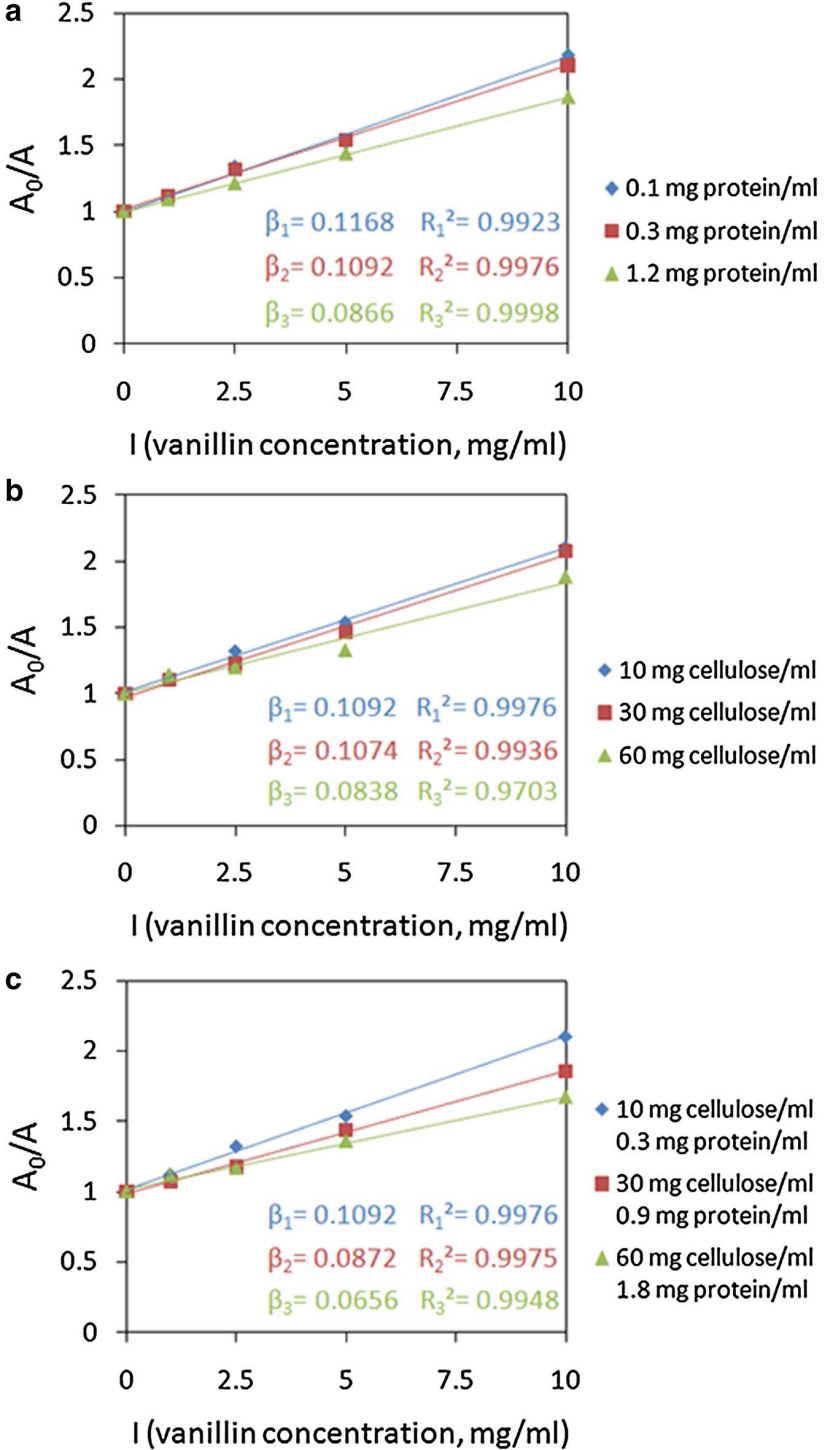
Linear relationship between *A*_*0*_/*A* and vanillin concentration. **a** Different cellulase concentrations (cellulose loading was 1 %); **b** Different cellulose loadings (cellulase concentration was 0.3 mg/ml); **c** Different cellulase concentrations and cellulose loadings

Previous study determined *β* values of several kinds of phenolics [29]. We found that the inhibition of vanillin was applicable to this equation as well, as shown in Fig. 4. The high relevance between (*A*_*0*_/*A*) and inhibitor concentration was observed. The value of *β* decreased as enzyme concentration and cellulose concentration increasing, suggesting that the binding ability between enzyme and inhibitor reduced.

**Fig. 5:**
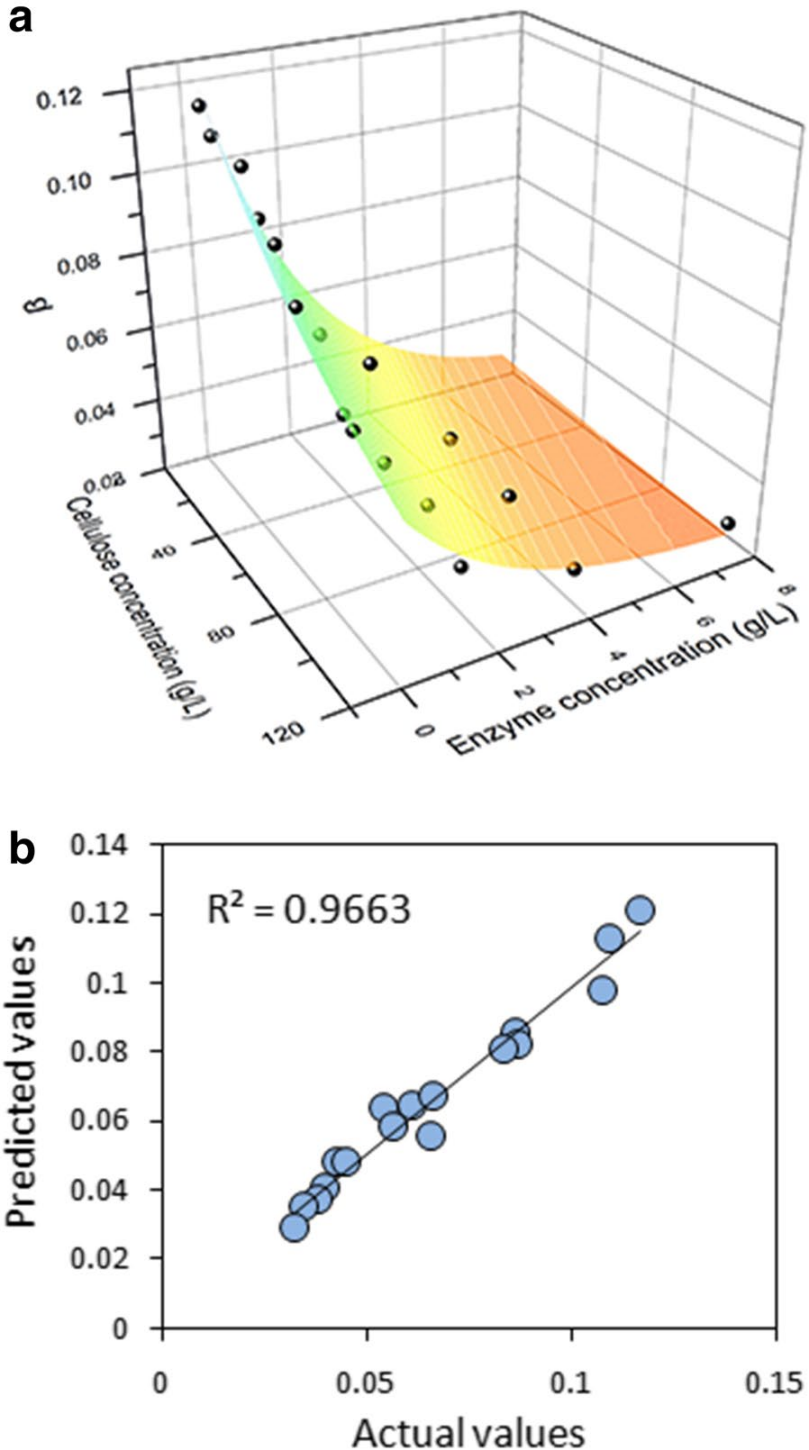
Curve fitting of *β* value. **a** Relationship between *β*, cellulose concentration and enzyme concentration; **b** Predicted *β* values versus actual *β* values using the exponential equation

According to the experimental data between cellulose concentration, enzyme concentration, and *β* values (Additional file 5: Table S1), we tried to develop an empirical model using nonlinear regression analysis. The exponential equation was the best fitting model, as shown in Fig. 5 and expressed by the following formula:

3 $$ \beta = \, 0.02788 \, + \, 0.1069 \, \times \, \exp \, ({-}0.009634S) \times \, \exp \, ({ - }0.4322E) $$

where *S* is the cellulose concentration (mg/mL) and *E* is the enzyme concentration (mg/mL).

As a result, the value of *A*_*0*_/*A* can be expressed as the function of cellulose, enzyme, and inhibitor concentration by substituting Eq. (3) into Eq. (2). The inhibition is positive correlation with inhibitor concentration and negative correlation with cellulose and enzyme concentrations. From Eqs. (2) and (3), we can see that the impact of inhibitor concentration on inhibition is much greater than cellulose and enzyme concentrations, because *A*_*0*_/*A* has linear relation with inhibitor concentration and is exponential rates-of-change with cellulose and enzyme concentration. As inhibitors are usually coupled with cellulose in the pretreated biomass, the inhibition increases as substrate loading increasing. It is notable that this relation is suitable when the enzyme is not excessive. Moreover, inhibition was determined with specific substrate, enzyme, and inhibitor at 50 °C and pH 4.8. The coefficients in Eq. (3) and the inhibition degree may vary with these conditions changing. Nonetheless, the linear relationship with inhibitor concentration and the exponential relationship with cellulose and enzyme concentration revealed the effects of cellulosic substrates, enzymes, and phenolics on inhibition.

### Effect of additives on inhibition

In order to reduce the inhibition of phenolics, calcium chloride, BSA, Tween 80, and activated carbon were added into the hydrolysate with the presence of vanillin (Table 1). Calcium chloride was proven to be effective at reducing lignin inhibition through the formation of lignin-metal complex [30]. BSA can attach lignin and significantly reduce non-specific adsorption of cellulase on lignin, leaving more cellulase free in solution [10, 31]. Tween series were also shown to enhance enzymatic digestibility of pretreated biomass [32, 33]. However, these additives did not improve the cellulose conversion in the absence and presence of vanillin. The BSA-pre-incubated trial exhibited no different cellulose conversions from the non-pre-incubated trial, which revealed that the effect of blocking agents on soluble inhibitors is negligible. The same to other additives, the results indicated that these kinds of additives would not be expected to have a major impact on the inhibition of soluble inhibitors. It also demonstrated that the inhibition mechanism of phenolics was different from insoluble lignin.

**Table 1:** Effect of additives on cellulose conversion^a^ and apparent inhibition degrees

Additives	Cellulose conversion without vanillin (%)	Cellulose conversion with vanillin (%)	Apparent inhibition (%)
Blank^b^	41.9 ± 0.5	26.8 ± 0.6	35.9 ± 0.6
CaCl_2_^b^	42.8 ± 0.2	27.0 ± 1.3	36.7 ± 0.5
Tween 80^b^	42.0 ± 1.5	28.5 ± 1.5	32.2 ± 1.5
Granular activated carbon^b^	39.7 ± 0.8	24.8 ± 1.4	37.5 ± 1.1
Powdered activated carbon^b^	28.6 ± 0.6	14.1 ± 1.7	50.5 ± 1.0
BSA^b^	42.0 ± 1.0	27.5 ± 0.3	34.6 ± 0.6
Blank^c^	41.9 ± 0.5	32.4 ± 1.0	22.6 ± 0.3
BSA^c^	40.8 ± 0.3	31.5 ± 0.3	22.8 ± 0.3
BSA^c, d^	40.8 ± 0.8	31.5 ± 1.0	23.2 ± 0.9

^a^Enzymatic hydrolysis of Avicel was carried out with cellulose concentration of 10 mg/mL and cellulase concentration of 0.3 mg/mL at 50 ºC. Cellulose conversion was determined at 24 h. The concentration of the additives was 2.5 mg/mL

^b^Vanillin concentration was 5 mg/mL

^c^Vanillin concentration was 2.5 mg/mL

^d^Vanillin was pre-incubated 3 h with BSA before enzyme addition

### Hypothesis of the interaction between phenolics, enzyme, and cellulose

**Fig. 6:**
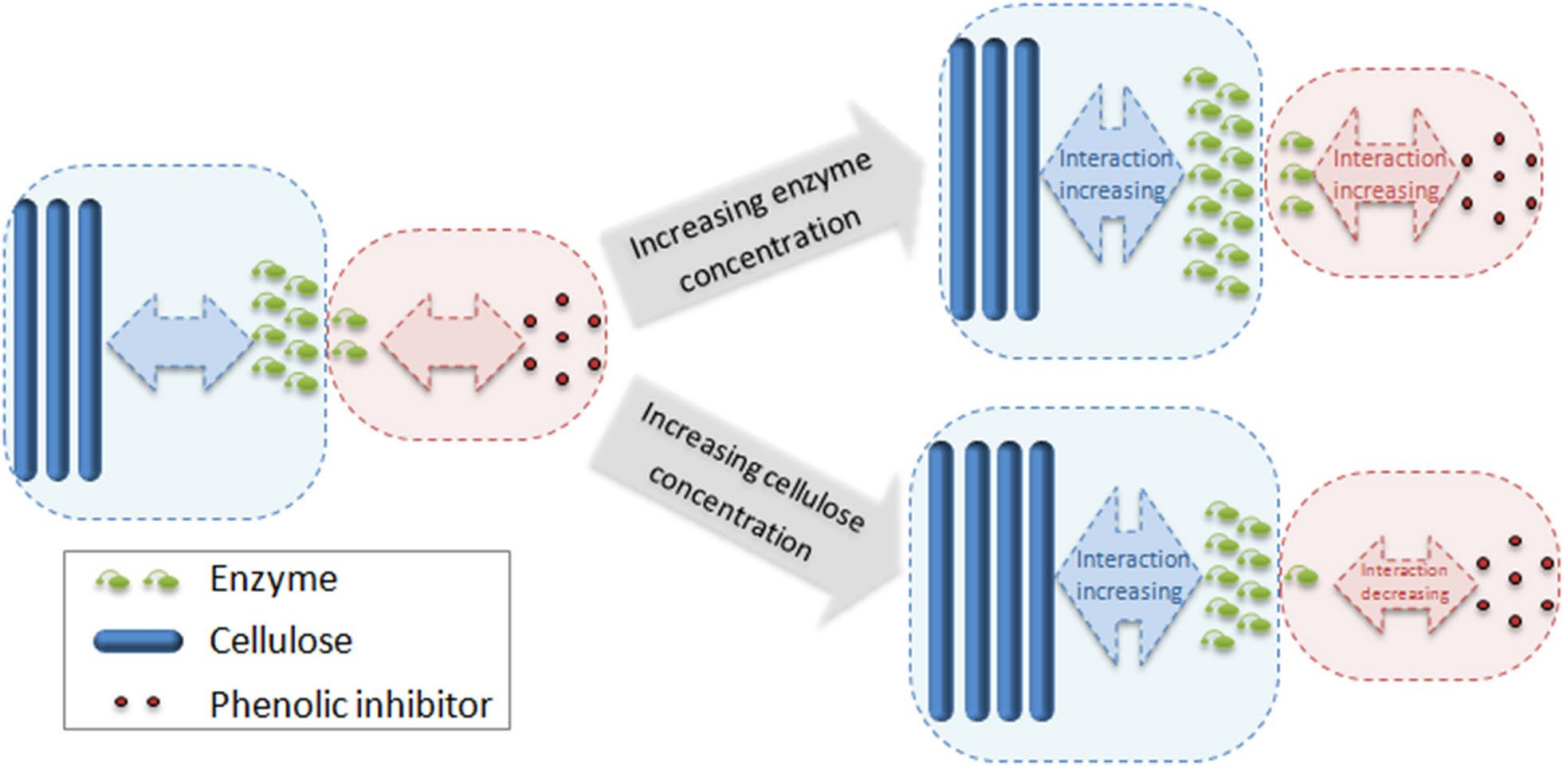
*Schematic diagram* of the interaction between enzyme, cellulose and phenolic inhibitor

The decrease of the inhibitor-binding constant is probably due to the higher affinity between enzyme and cellulose than that between enzyme and inhibitor. When enzyme concentration increased, more additional enzyme reacted with cellulose, and less additional enzyme was deactivated by phenolic inhibitor, causing the proportion of deactivated enzyme reduced comparing to original enzyme loading. Likewise, increasing cellulose concentration increased the proportion of active enzyme to cellulose and reduced the proportion of deactivated enzyme. Thus, inhibitor-binding ability was decreased comparing to former conditions (Fig. 6). Previous study showed that phenolics concentration in hydrolysate was also consumed with the precipitation of enzymes, supporting phenolics and enzymes are tightly bonded. A high ratio of 25 mg protein/mg phenolics can exhaust phenolics gradually [17]. It was reported that the inhibition of macromolecular lignin in steam pretreated biomass can be overcome by increasing enzyme concentration [27]. The difference between inhibition of phenolics and inhibition of lignin is supposed to be as follows: the adsorption sites of macromolecular lignin are limited and can be saturated by increasing protein concentration, resulting in elimination of inhibition; in contrast, the inhibition of soluble phenolics can hardly be entirely removed by increasing enzyme concentration or adding blocking protein due to the dispersity and multiple binding sites of phenolics, which was proved by the BSA addition test. Hence, other detoxification methods are needed to overcome phenolic inhibitors.

### Effect of pH and temperature on inhibition

Inhibition of vanillin was determined at different pH (4.3-5.3) and different temperatures (30–50 °C) in enzymatic hydrolysis. Increasing pH slightly released inhibition_app_ (Table 2). Glucan conversion with inhibitor addition kept at ~27 % at three pH values. Inhibition_app_ reduced only from 35.7 to 32.6 % when pH increased from 4.3 to 5.3, and the corresponding inhibitor-binding constant reduced from 0.111 to 0.097. The elevated pH decreased nonspecific cellulase binding to lignin by increasing lignin surface charge and hydrophilicity [34]. The slight decrease of vanillin inhibition in this study may be caused by the potential-induced change of inhibitor-binding capacity, which needs to be studied further.

**Table 2:** Effect of pH on vanillin inhibition

PH	Cellulose conversion^a^ without vanillin (%)	Cellulose conversion with vanillin^b^ (%)	Apparent inhibition (%)	Inhibitor-binding constant *β* (vanillin, mL/mg)
4.3	40.9 ± 2.1	26.3 ± 1.8	35.7 ± 2.8	0.1112
4.8	41.4 ± 0.6	26.9 ± 0.6	35.0 ± 0.9	0.1092
5.3	39.5 ± 2.0	26.6 ± 0.3	32.6 ± 2.0	0.0967

^a^Enzymatic hydrolysis of Avicel was carried out with cellulose concentration of 10 mg/mL and cellulase concentration of 0.3 mg/mL at 50 ºC. Cellulose conversion was determined at 24 h

^b^Vanillin concentration was 5 mg/mL

Changing temperature hardly alleviated inhibition of vanillin (Table 3). When hydrolysis temperature increased from 30 to 50 °C, glucan conversions without inhibitor increased from 22.0 to 41.4 % and glucan conversions with inhibitor increased from 14.4 to 26.9 %. However, the corresponding inhibition_app_ and inhibitor-binding constant were almost unchanged.

**Table 3:** Effect of temperature on vanillin inhibition

Temperature (ºC)	Cellulose conversion^a^ without vanillin (%)	Cellulose conversion with vanillin^b^ (%)	Apparent inhibition (%)	Inhibitor-binding constant *β* (vanillin, mL/mg)
30	22.0 ± 1.7	14.4 ± 0.2	34.4 ± 3.0	0.1048
40	35.7 ± 0.6	22.9 ± 0.5	35.9 ± 1.6	0.1121
50	41.4 ± 0.6	26.9 ± 0.6	35.0 ± 0.9	0.1092

^a^Enzymatic hydrolysis of Avicel was carried out with cellulose concentration of 10 mg/mL and cellulase concentration of 0.3 mg/mL at pH 4.8. Cellulose conversion was determined at 24 h

^b^Vanillin concentration was 5 mg/mL

### Effect of chemical groups on inhibition

**Fig. 7:**
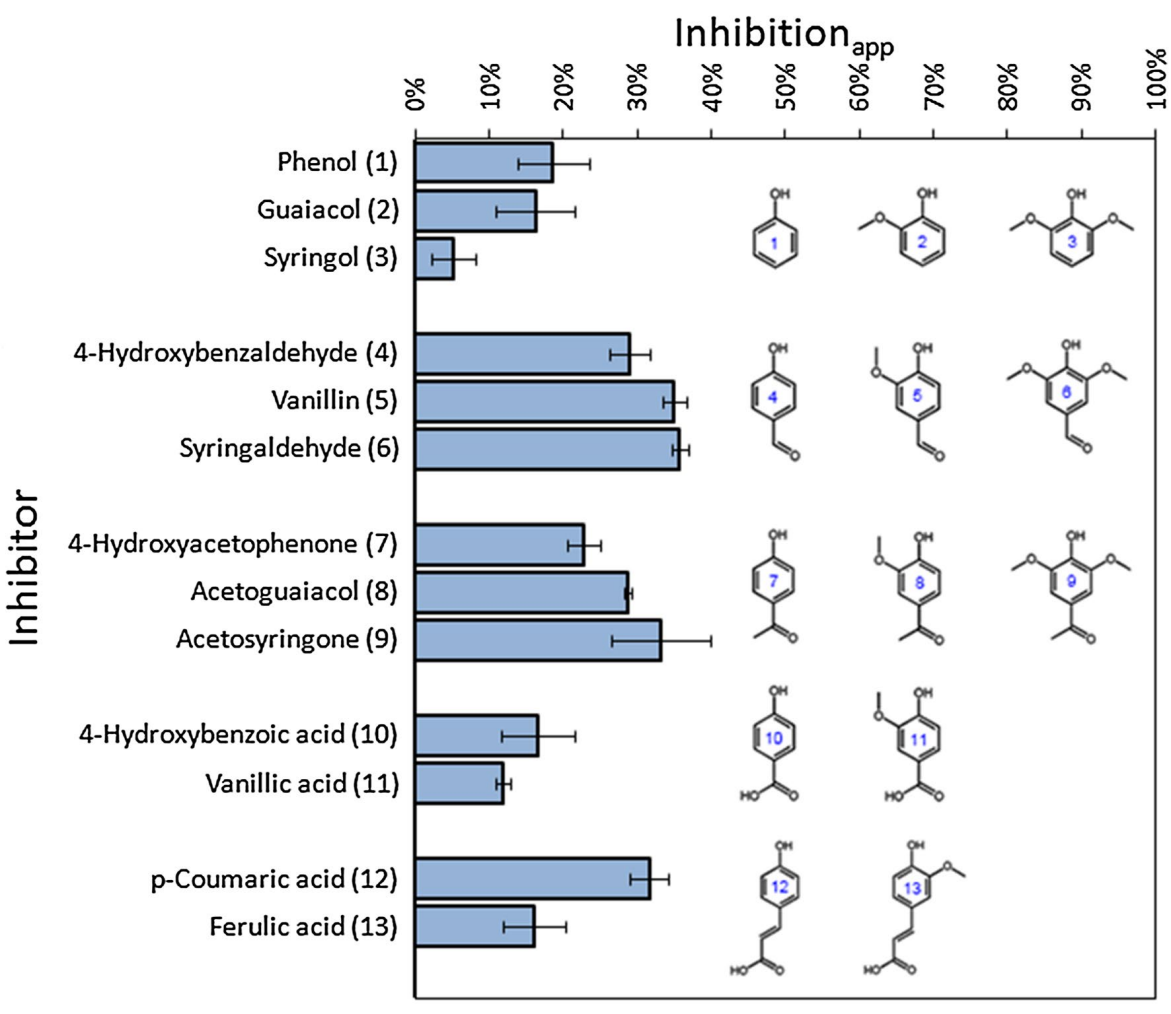
Apparent inhibitions of different phenolic compounds. Phenolic concentrations are all 5 mg/mL. *Error bars* represented standard deviations, *n* = 3

We also investigated the inhibition of different phenolics compounds in enzymatic hydrolysis (Fig. 7). All the phenolic compounds inhibited cellulose conversion and exhibited different inhibition_app_. Inhibition_app_ decreased as the adjunction of methoxy side chain at C2 and C6 for phenols, such as 4-hydroxybenzoic acids and *p*-coumaric acid. However, the increased inhibition_app_ was observed as the adjunction of methoxy side chain for 4-hydroxybenzaldehyde and 4-hydroxyacetophenone. For the specific aromatic unit (H, G, and S), compounds with aldehyde and ketone group exhibited more inhibitory effect on cellulase than those with phenol and carboxyl group. It was reported that total hydroxyl groups in lignin had an important impact on cellulase adsorption and enzymatic hydrolysis [35]. Our results indicated that carbonyl group and methoxy group in phenolics also inhibit enzyme activity.

## Conclusion

Vanillin significantly decreased enzyme activity and concentration during enzymatic hydrolysis. The inhibition degree (*A*_*0*_/*A*) was independent of the hydrolysis time. The increasing of cellulase concentration or cellulose loading slightly mitigated the inhibition of vanillin. The inhibition degree depended linearly on the inhibitor concentration and exponentially on the cellulose loading and the enzyme concentration. pH, temperature, and the addition of calcium chloride, BSA and Tween 80 cannot mitigate the inhibition of vanillin significantly. The presence of hydroxyl group, carbonyl group, and methoxy group in phenolics exhibited the impact on inhibitions.

## Experimental

### Materials

Avicel PH-101 (containing 98 % glucan and 2 % xylan in dry matter) purchased from Sigma-Aldrich (St. Louis, MO) was used as cellulosic substrate. The use of pure cellulosic substrate is in order to exclude the interference of other compositions in lignocellulosic material.

Commercial cellulase Spezyme CP preparation from *Trichoderma reesei* containing exo-, endo-, and *β*-glucosidase activities was provided by Genencor (Palo Alto, CA), which has a total protein concentration of 123 mg/mL. The cellulase activity was determined as 59 FPU/mL.

Phenol, guaiacol, syringol, 4-hydroxybenzaldehyde, vanillin, syringaldehyde, 4-hydroxyacetophenone, acetoguaiacol, acetosyringone, 4-hydroxybezoic acid, and vanillic acid were purchased from Sigma-Aldrich (St. Louis, MO). *p*-Coumaric acid and ferulic acid were purchased from Aladdin (Los Angels, CA).

### Enzymatic hydrolysis

Enzymatic hydrolysis was conducted with 20-mL reaction volume in 100-mL flask, with cellulase at 50 °C and 150 rpm. Avicel, phenolics, and cellulase were added to specific concentration or loading. 50 mM citrate buffer (pH 4.8) and 20 mg/L sodium azide were used in enzymatic hydrolysis. 0.2 mL samples were withdrawn and frozen at −20 °C for subsequent HPLC sugar analysis.

### Analytical methods

The National Renewable Energy Laboratory (NREL) LAP-004 protocol was used to determine the composition of Avicel. Glucose was analyzed by HPLC equipped with an Aminex HPX-87H column (Bio-rad, Hercules, CA) at 65 °C.

The protein concentrations of the enzymes were determined by the BCA protein assay.

## Additional files

10.1186/s13068-016-0485-2 Cellulose hydrolysis rate with different vanillin concentrations. Cellulose loading was 1% (10 mg/mL) and cellulase concentration was 0.3 mg/mL.
10.1186/s13068-016-0485-2 Enzyme concentration in supernatant. wo: without; w: with; I: inhibitor; S: substrate. Vanillin concentration was 5 mg/mL. Error bars represented standard deviations, n=2.
10.1186/s13068-016-0485-2 Enzyme activity decreased as enzyme pre-incubation time went without (A) and with (B) 5 mg/mL vanillin addition. Enzyme activity was calculated by glucose concentration in hydrolysate within one hour. Cellulose loading was 1% and cellulase concentration was 0.3 mg/mL. The enzyme activity of 0 h pre-incubation without vanillin addition was standardized as 100 %. Error bars represented standard deviations, n=2.
10.1186/s13068-016-0485-2 Cellulose conversions (A) and inhibition of vanillin (B) with different vanillin loadings at 24 h of hydrolysis. Error bars represented standard deviations, n=3.
10.1186/s13068-016-0485-2Inhibitor-binding constants of different cellulose and cellulose concentrations.

## Abbreviations

H: hydroxyphenyl unit
G: guaiacyl unit
S: syringyl unit
Inhibition_app_: apparent inhibition degree
*A*: cellulose conversion with inhibitor
*A*_*0*_: cellulose conversion without inhibitor
*I*: concentration of inhibitor (g/L)
*β*: inhibitor-binding constant (L/g)
*S*: cellulose concentration (mg/mL)
*E*: enzyme concentration (mg/mL)
BSA: bovine serum albumin
HPLC: high performance liquid chromatography
FPU: filter paper unit
