# Supplementary material for: Inhibition of lignin-derived phenolic compounds to cellulase
Source: Biotechnol Biofuels. 2016 Mar 22;9:70. doi: 10.1186/s13068-016-0485-2 (PMC4802812; doi:10.1186/s13068-016-0485-2)
Supplement: Supplementary file 1 — 10.1186/s13068-016-0485-2 Cellulose hydrolysis rate with different vanillin concentrations. Cellulose loading was 1% (10 mg/mL) and cellulase concentration was 0.3 mg/mL. [file 13068_2016_485_MOESM1_ESM.docx]

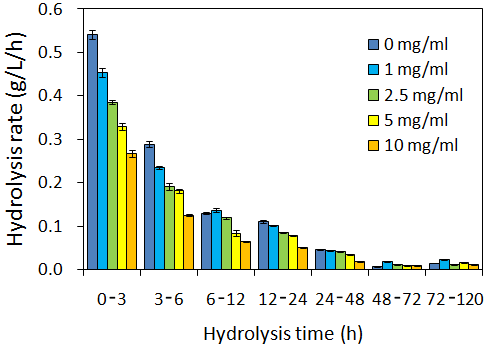


**Figure S1 Cellulose hydrolysis rate with different vanillin concentrations.** Cellulose loading was 1% (10 mg/mL) and cellulase concentration was 0.3 mg/mL.
