# Supplementary material for: Inhibition of lignin-derived phenolic compounds to cellulase
Source: Biotechnol Biofuels. 2016 Mar 22;9:70. doi: 10.1186/s13068-016-0485-2 (PMC4802812; doi:10.1186/s13068-016-0485-2)
Supplement: Supplementary file 2 — 10.1186/s13068-016-0485-2 Enzyme concentration in supernatant. wo: without; w: with; I: inhibitor; S: substrate. Vanillin concentration was 5 mg/mL. Error bars represented standard deviations, n=2. [file 13068_2016_485_MOESM2_ESM.docx]

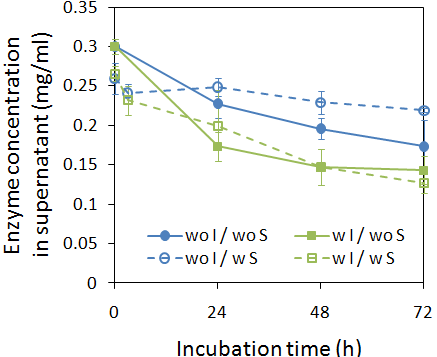


**Figure S2 Enzyme concentration in supernatant.** wo: without; w: with; I: inhibitor; S: substrate. Vanillin concentration was 5 mg/mL. Error bars represented standard deviations, n=2.
