# Supplementary material for: Inhibition of lignin-derived phenolic compounds to cellulase
Source: Biotechnol Biofuels. 2016 Mar 22;9:70. doi: 10.1186/s13068-016-0485-2 (PMC4802812; doi:10.1186/s13068-016-0485-2)
Supplement: Supplementary file 3 — 10.1186/s13068-016-0485-2 Enzyme activity decreased as enzyme pre-incubation time went without (A) and with (B) 5 mg/mL vanillin addition. Enzyme activity was calculated by glucose concentration in hydrolysate within one hour. Cellulose loading was 1% and cellulase concentration was 0.3 mg/mL. The enzyme activity of 0 h pre-incubation without vanillin addition was standardized as 100 %. Error bars represented standard deviations, n=2. [file 13068_2016_485_MOESM3_ESM.docx]

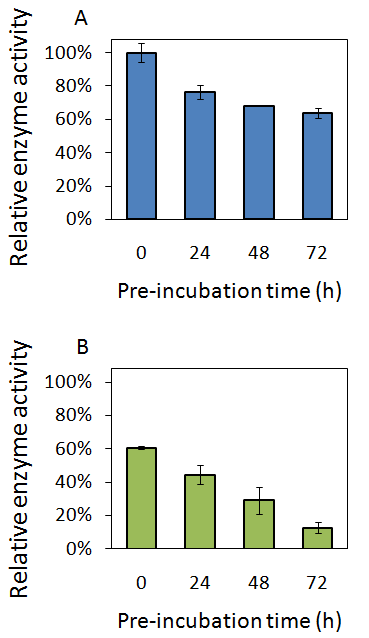


**Figure S3 Enzyme activity decreased as enzyme pre-incubation time went without (A) and with (B) 5 mg/mL vanillin addition.** Enzyme activity was calculated by glucose concentration in hydrolysate within one hour. Cellulose loading was 1% and cellulase concentration was 0.3 mg/mL. The enzyme activity of 0 h pre-incubation without vanillin addition was standardized as 100%. Error bars represented standard deviations, n=2.
