# Supplementary material for: Inhibition of lignin-derived phenolic compounds to cellulase
Source: Biotechnol Biofuels. 2016 Mar 22;9:70. doi: 10.1186/s13068-016-0485-2 (PMC4802812; doi:10.1186/s13068-016-0485-2)
Supplement: Supplementary file 4 — 10.1186/s13068-016-0485-2 Cellulose conversions (A) and inhibition of vanillin (B) with different vanillin loadings at 24 h of hydrolysis. Error bars represented standard deviations, n=3. [file 13068_2016_485_MOESM4_ESM.docx]

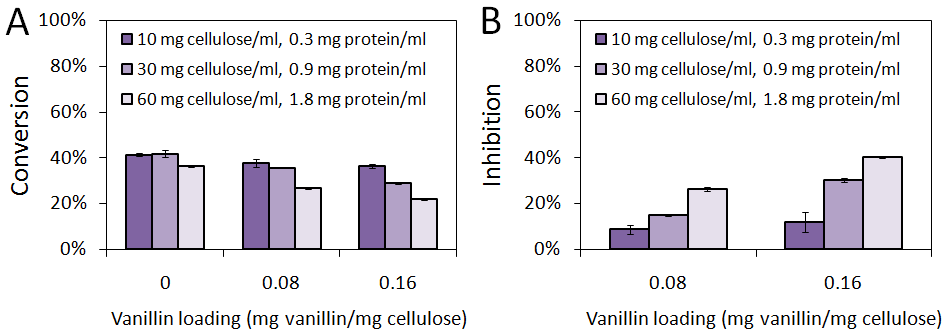


**Figure S4 Cellulose conversions (A) and inhibition of vanillin (B) with different vanillin loadings** **at 24 h of hydrolysis.** Error bars represented standard deviations, n=3.
