# Supplementary material for: Inhibition of lignin-derived phenolic compounds to cellulase
Source: Biotechnol Biofuels. 2016 Mar 22;9:70. doi: 10.1186/s13068-016-0485-2 (PMC4802812; doi:10.1186/s13068-016-0485-2)
Supplement: Supplementary file 5 — 10.1186/s13068-016-0485-2Inhibitor-binding constants of different cellulose and cellulose concentrations. [file 13068_2016_485_MOESM5_ESM.docx]

**Table S1** Inhibitor-binding constants of different cellulose and cellulose concentrations.

| No. | Cellulose concentration (mg/mL) | Enzyme concentration (mg/mL) | Inhibitor-binding constant *β* (vanillin, mL/mg)^a^ |
| --- | --- | --- | --- |
| 1 | 10 | 0.1 | 0.1168 |
| 2 | 10 | 0.3 | 0.1092 |
| 3 | 10 | 1.2 | 0.0866 |
| 4 | 30 | 0.3 | 0.1074 |
| 5 | 30 | 0.9 | 0.0872 |
| 6 | 30 | 1.8 | 0.0614 |
| 7 | 60 | 0.3 | 0.0838 |
| 8 | 60 | 1.8 | 0.0656 |
| 9 | 60 | 1.2 | 0.0544 |
| 10 | 60 | 3.6 | 0.0399 |
| 11 | 90 | 0.3 | 0.0664 |
| 12 | 90 | 0.9 | 0.0566 |
| 13 | 90 | 1.8 | 0.0427 |
| 14 | 90 | 3.6 | 0.0384 |
| 15 | 120 | 1.2 | 0.0451 |
| 16 | 120 | 3.6 | 0.0348 |
| 17 | 120 | 7.2 | 0.0324 |

^a^*β* was determined at 24 h dydrolysis.
